# Focal Lesions in Fatty Liver: If Quantitative Analysis Facilitates the Differentiation of Atypical Benign from Malignant Lesions

**DOI:** 10.1038/srep18640

**Published:** 2016-01-04

**Authors:** Quan-Yuan Shan, Li-Da Chen, Lu-Yao Zhou, Zhu Wang, Guang-Jian Liu, Yang Huang, Wei Li, Jin-ya Liu, Xiao-yan Xie, Ming-de Lu, Jie Liu, Wei Wang

**Affiliations:** 1 Department of Medical Ultrasonics, Institute of Diagnostic and Interventional Ultrasound, The First Affiliated Hospital of Sun Yat-Sen University, No. 58 Zhongshan Road 2, Guangzhou, China; 2 Department of Medical Ultrasonics, Institute of Diagnostic and Interventional Ultrasound, The Sixth Affiliated Hospital of Sun Yat-Sen University, No. 26 Yuancun’erheng road, Guangzhou, China; 3 Department of Hepatobiliary Surgery, The First Affiliated Hospital of Sun Yat-Sen University, No. 58 Zhongshan Road 2, Guangzhou, China; 4 Department of Biomedical Engineering, School of Engineering, Sun Yat-sen University, No. 135 Xinxiang West Road, Guangzhou, Guangdong, China

## Abstract

To evaluate the diagnostic performance of quantitative analysis as an adjunctive diagnostic tool to contrast-enhanced ultrasound (US) for the differentiation of atypical benign focal liver lesions (FLLs) from malignancies in fatty liver. Twenty-seven benign FLLs and fifty-six malignant FLLs that appeared hyper-enhanced during the arterial phase with washout in the portal or late phase in fatty liver were analyzed. Chi-square tests and logistic regression were applied to identify the specific features. Three sets of criteria were assigned: 1) all FLLs subjected to routine contrast-enhanced US; 2) all FLLs subjected to quantification analysis and contrast-enhanced US; and 3) parts of FLLs that could not be diagnosed using contrast-enhanced US (n = 66, 75.9%) but instead were diagnosed using parametric features. The sensitivity, specificity, accuracy and area under the receiver operating characteristic curve (AUC) of the three sets of criteria were analyzed. The AUCs of the **c**riterion set 2 were significantly higher than those of **c**riterion set 1 (0.904 versus 0.792, P = 0.008). **C**riterion set 3 showed a relatively high sensitivity (90.2%) with a relatively high AUC (0.845). The quantification analysis offers improved diagnostic performance for the differential identification of atypical benign FLLs from malignancies in fatty liver.

In contrast to the appearance in normal liver on conventional ultrasound^1^, the percent of hypoechoic benign focal liver lesions (FLLs) increases in fatty liver, and some benign focal liver lesions in fatty liver present isoechoic or slight hyperechoic^2^,^3^,^4^. In addition, benign signs, such as posterior echo enhancement, are also frequently detected in metastatic lesions due to fatty changes^2^,^4^. The diagnostic dilemma caused by the phenomenon above regarding conventional ultrasound has been markedly improved by contrast-enhanced US^3^,^5^. However, 4.9%–21.4% of benign FLLs show washout in the portal or late phase in contrast-enhanced US^3^,^6^,^7^. Such benign FLLs are misdiagnosed as malignancies, especially for those without typical imaging features in the arterial phase. To date, few reports have focused on this challenging and critical issue regarding fatty liver.

Quantitative analysis of contrast-enhanced US images allows for an objectively accurate assessment of blood-flow kinetics within tumors^8^,^9^,^10^,^11^,^12^,^13^,^14^,^15^. Although the visual interpretation is largely dependent on the observer’s experience, quantification has been used to overcome subjective evaluation of the enhancement between a focal lesion and the surrounding tissue^9^. It has been reported that classic malignant FLLs may receive blood supply from arteries, whereas benign FLLs are supplied by both portal tracts and arteries^16^,^17^, implying that malignant FLLs would sustain greater enhancement than benign FLLs. In addition, based on our preliminary studies^6^, malignant FLLs would washout more rapidly without the blood supply of the portal vein. Therefore, we propose that the diagnostic performance of contrast-enhanced US for differentiating among FLLs in fatty liver would be improved by quantification.

The purpose of our study was to retrospectively evaluate the diagnostic performance of quantitative analysis as an adjunctive diagnostic tool to routine contrast-enhanced US for the identification of benign FLLs that mimic malignant FLLs in fatty liver background.

## Materials and Methods

### Patient population

**Figure 1:**
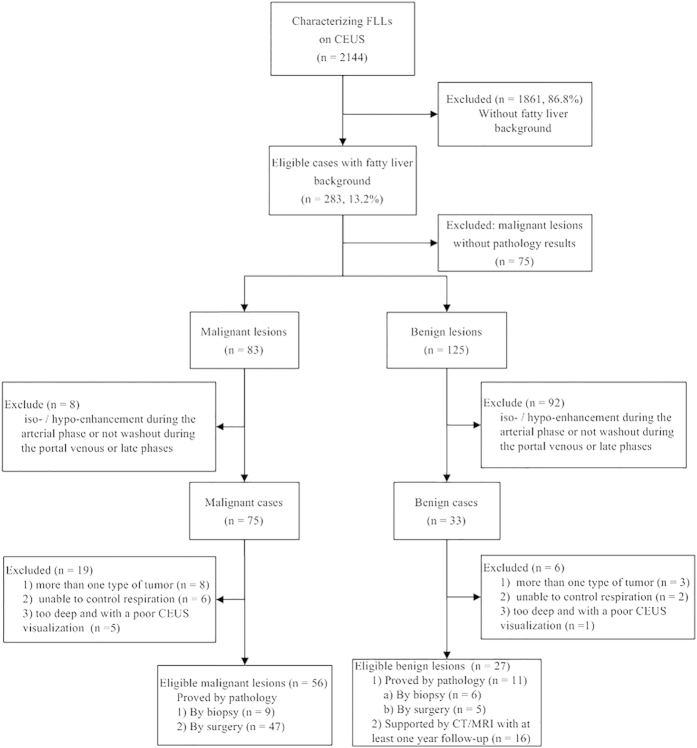
Flow diagram of the study population.

This retrospective study was approved by the first affiliated hospital of Sun Yat-Sen University Institutional Review Board, and informed consent was waived. We designed the study and we report it according to the STARD (standards for reporting of diagnostic accuracy) criteria. All experiments were performed in accordance with the relevant guidelines and regulations. Between January 2012 and October 2014, a total of 2,144 patients were referred to our department to receive contrast-enhanced US examination for characterization of FLLs. Among the FLL patients, there were 283 patients with a background of a fatty liver, including 158 malignant lesions and 125 benign lesions. In total, 83 cases that were diagnosed as eligible malignant or as benign lesions were ultimately included in the study based on the inclusion and exclusion criteria (Fig. 1). Patients were included if they met the following inclusion criteria: (a) the echogenicity of the liver parenchyma was evaluated as fatty liver, which was confirmed by pathology or by CT/MRI; (b) they had lesions visually exhibiting hyperenhancement during the arterial phase and, later, during washout in the contrast-enhanced US; (c) the lesions were confirmed as malignant based on pathology or as benign lesions either based on pathology or supported by CT/MRI with at least a one-year follow-up; and (d) no biopsies or treatments were conducted before the contrast-enhanced US. The exclusion criteria were as follows: (a) the patient had more than one type of tumor; (b) the patient had uncontrollable respiration with regard to quantitative analysis; or (c) the patient had a deep FLL for which contrast-enhanced US visualization was too poor for quantitative analysis (Fig. 1). If patients exhibited more than one lesion, the largest and most clearly displayed lesion was selected for evaluation. The data from 11 HCC cases and 3 FNH cases overlapped with those of patients reported in previous studies^18^,^19^. However, these studies have no other data in common; different analyses were conducted, and new results were obtained in the present study.

Among the 83 patients (62 male and 21 female), there were 56 malignant cases and 27 benign cases (overall mean age, 50.2 years; range, 21–79 years). The mean age of the male patients (mean age, 49.9 years; range, 21–76 years) did not significantly differ from that of the female patients (mean age, 52.5 years; range, 31–79 years) (P = 0.383). Among the 56 malignant lesions, the diagnosis was confirmed via pathological analysis of the specimens obtained from US-guided percutaneous biopsies (n = 9) or surgical resections (n = 47). Of the 27 benign lesions, 11 were confirmed based on pathology (6 by biopsy and 5 by surgical resection), and the other 16 diagnoses were supported by clinical follow-ups. The diagnoses of malignancy upon histologic examination included hepatocellular carcinoma (n = 34), cholangiocarcinoma (n = 8), and metastasis (n = 14). The diagnoses of benign lesions upon histologic examination included focal nodular hyperplasia (n = 9), abscess (n = 5), hemangioma (n = 3), inflammatory pseudotumor (n = 3), focal fatty change (n = 3), parasite-caused infection (n = 2), epithelioid angiomyolipoma (n = 1), and hemangioblastoma (n = 1).

### Examination technique

Ultrasound examinations were performed using an Aplio SSA-770A (n = 55) or an Aplio 500 (n = 28) (Toshiba Medical Systems, Tokyo, Japan) scanner equipped with a 375 BT convex transducer (frequency range, 1.9 to 6.0 MHz). Harmonic contrast imaging was performed with a mechanical index that ranged from 0.06 to 0.10. The transmission frequency used for CHI was 3.0 or 3.5 MHz. Conventional US was performed to scan the liver thoroughly before the contrast-enhanced US examination. In addition, ample identification and observation of the target lesions upon baseline investigation in B-mode was necessary. The imaging settings, such as gain, depth, and focus, were optimized. Generally, a single focus was placed at the bottom of the lesion. CHI was initiated with a stable transducer position. After activating CHI mode, a bolus injection of 2.4 ml of *SonoVue*^*®*^ (Bracco Suisse SA, Switzerland) was administered intravenously via an antecubital vein, followed immediately by a flushing with 5 ml of normal saline solution. The targeted lesion was observed continuously for 5 minutes. Consecutive 60-second cine clips were recorded beginning at the wash-in to demonstrate the arterial features through the peak of enhancement, and the portal venous phases and several repetitions of the late phase were stored on a hard disk. The arterial, portal venous and late phases were defined as 0–30 second(s), 31–120 s and 121–360 s after the injection, respectively. Contrast-enhanced US examinations were performed by one of three radiologists (X.Y.X., Z.W. and W.W.) experienced with routine US (28, 11 and 10 years of experience, respectively) and contrast-enhanced US (15, 8 and 6 years, respectively).

### Image analysis and contrast-enhanced US features

The contrast-enhanced US images were reviewed by two staff radiologists (L.D.C. and L.Y. Z.), each with at least 4 years of experience evaluating liver contrast-enhanced US and at least 10 years of experience evaluating liver US. The reviewers initially read the images independently; if there was any disagreement, the images were re-reviewed, and a determination was reached by consensus. None of the readers had been involved in the original examinations, and all were blinded to the patients’ identities, clinical histories, other imaging findings and pathological results. In each case, for the target lesion, size, depth, number of nodules, echogenicity, shape and margin in conventional ultrasound and the enhancement intensity and pattern during each vascular phase were recorded. The size of lesion was the major diameter measured in the maximum section. Depth was the deepest edge of the lesion to the surface. Echo of the lesion was compared with liver background and was divided into hypo-, iso-, hyper- and mixed echogenicity.

The diagnostic criteria for malignant and benign lesions were determined according to the 2012 European Federation of Societies for Ultrasound in Medicine and Biology Guidelines^20^. Each group used a subjective five-point scale to grade diagnostic confidence (grade 1 = definitely benign lesions; grade 2 = most likely benign lesions; grade 3 = indeterminate; grade 4 = most likely malignant lesions; grade 5 = definitely malignant lesions). According to tables in the guideline^20^, the diagnostic features for malignant lesions included the following: rim-enhancement, and/or with complete enhancement, hyperenhancement, or nonenhancing regions; hyperenhancing, and/or with nonenhancing regions; rim-like hyperenhancement, central hypoenhancement, and/or with nonenhancing regions, inhomogeneous, or hyperenhancement; hyperenhancing, complete nonenhancing areas (if large), and/or with enhancing tumor thrombus or hypo/nonenhancing during the arterial phase. The diagnostic features for benign lesions included the following: peripheral nodular enhancement, or small lesion with complete, rapid centripetal enhancement; hyperenhancing from the center, complete, early, and/or with spoke-wheel arteries or feeding artery; hyperenhancing, complete, and/or with nonenhancing regions; isoenhancing; peripheral enhancement, no central enhancement, and/or with enhanced septa or hyperenhanced liver segment during the arterial phase.

For each patient, the level of fatty liver was classified into one of three grades as follows: Grade I (mild) = mildly diffuse increase in liver echogenicity and clear visualization of the diaphragm and intrahepatic vessel walls; Grade II (moderate) = moderate grade diffuse increase in liver echogenicity obscuring the intrahepatic vessel walls and the diaphragm; Grade III (severe) = prominent liver echogenicity increment in liver echogenicity and poor or nonvisualization of the hepatic vessels and diaphragm^3^.

### Quantitative imaging analysis with contrast-enhanced US

The image analysis obtained still frames from the contrast-enhanced US cine clips (from 0 s to 360 s) of all 56 malignant FLLs and 27 benign FLLs records, and all images were transformed into an 8-bit tag image file format. Contrast-enhanced US images were further analyzed using the free, public domain Java-based image processing tool, ImageJ software (Wayne Rasband, National Institutes of Health, Bethesda). ImageJ was downloaded from http://www.rsb.info.nih.gov/ij/, which can be used to calculate area and pixel value statistics of regions of interest (ROIs). To avoid interobserver variability, all parametric imaging was performed by one of the investigators (Q.Y.S.) who had no access to any identifying information of the patients. To avoid recall bias, the operator who performed the quantitative analysis did not participate in patient care. The inter-observer variability of the selected parameters for each patient was performed independently by a second operator (G.J.L.). All quantification analyses were performed off-line in a single session, and both operators were blinded to clinical outcome of the patients. The time interval between the quantitative analysis and contrast-enhanced US image evaluation was greater than 2 months. A borderline ROI was drawn to select the area to be analyzed. An ROI was drawn along the margin of each lesion and excluded the non-enhanced area, and a reference ROI was defined as the peripheral parenchyma at a depth similar to that of the lesion, avoiding any extra vessels or regions with excessive US attenuation.

**Figure 2:**
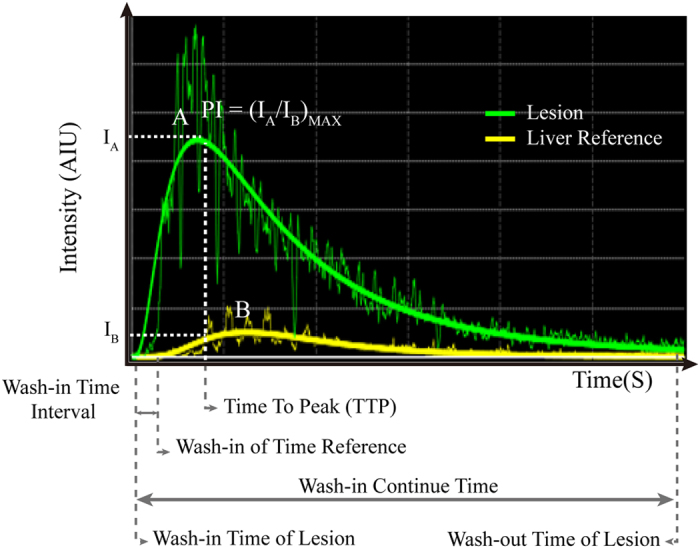
Diagram of time–intensity curve.

The parameters obtained using the software included the following: wash-in time to the lesion—the time from the injection of the contrast agent to its arrival at the lesion; wash-in time to the reference—the time from the injection of the contrast agent to its arrival at the reference; wash-in time interval—the time interval between the wash-in time to the lesion and to the reference; peak intensity (PI)—the percentage ratio of the intensity of the ROI in the lesion to the ROI in the reference at the highest points of the perfusion processes; time to peak (TTP)—the time from the contrast’s arrival at the lesion to the peak enhancement intensity; washout time—the time from the injection of the contrast agent to its washout from the lesion; wash-in continue time—the time from the contrast’s arrival at the lesion to the time of its washout from the lesion (i.e., the time interval between the wash-in time and the washout time for the lesion). All the parameters were marked in the diagram of the time–intensity curve (Fig. 2).

### Statistical analysis

**Figure 3:**
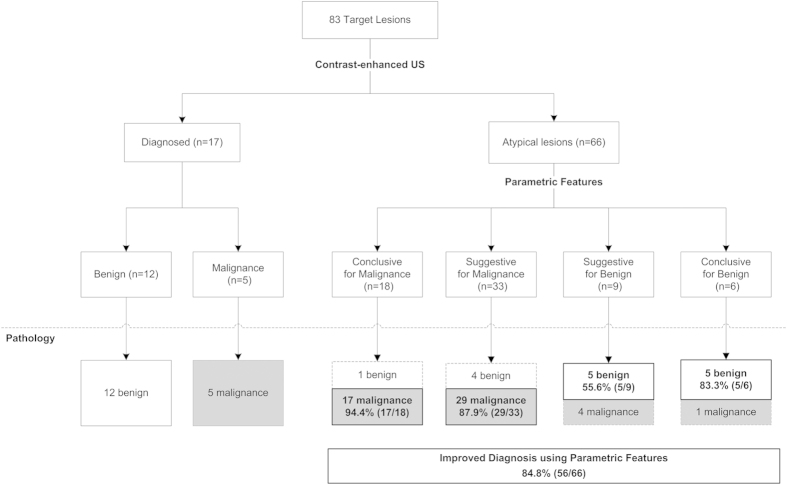
Flow diagram of the representation of the appearance of the atypical benign focal liver lesions that could not be diagnosed using contrast-enhanced ultrasound, which were instead diagnosed using parametric features.

All quantitative data are reported as the means ± SDs. Paired t-tests were used to compare quantitative parameters of the lesions and the related peripheral reference tissues. Independent-sample t-tests were used to compare quantitative parameters of the malignant lesions and the benign lesions. Chi-squared tests were used to evaluate the differences between among the qualitative data. Typical features were selected to determine their associations with the malignant lesions and the benign lesions using logistic regression analysis. To assess the inter-observer variability, the intraclass correlation coefficient (ICC) was used. The ICC was categorized as follows: slight, 0 ≤ ICC < 0.20; fair, 0.21 ≤ ICC < 0.40; moderate, 0.41 ≤ ICC < 0.60; substantial, 0.61 ≤ ICC < 0.80; and almost perfect, ICC > 0.81. The lesions assigned confidence rating scores of 3, 4 or 5 were regarded as malignant lesions, and those assigned confidence rating scores of 1 or 2 were defined as benign lesions. To validate whether adding parametric features to contrast-enhanced US improved the diagnostic performance in predicting malignant lesions versus benign lesions, the following three sets of criteria were assigned: criterion set 1—liver lesions diagnosed from routine contrast-enhanced US features; criterion set 2—liver lesions diagnosed using parametric features and contrast-enhanced US; and criterion set 3—liver lesions that could not be distinguished using contrast-enhanced US in criterion set 1 (n = 66, 75.9%) (Fig. 3) diagnosed using parametric features. The parametric features contained all of the significant features in the preceding analysis. Sensitivity, specificity and accuracy were calculated. Receiver operating characteristic curves were plotted to evaluate the diagnostic performance of the three criteria sets for discriminating between malignant lesions and benign lesions. The diagnostic performance was expressed as the area under the ROC curve (AUC). Differences in the AUCs were assessed using the method described by Hanley and McNeil^21^. P < 0.05 indicated a statistically significant difference. The statistical analyses were performed using the statistical software MedCalc version 10.2.0.0 (MedCalc Software, Mariakerke, Belgium) and SPSS13.0 (SPSS Inc., Chicago, USA).

## Results

### Clinical characteristics, conventional US characteristics and contrast-enhanced US features

The mean age of the patients with malignant nodules (mean age, 52.3 years; range, 21–79 years) was greater than that of the patients with benign nodules (mean age, 45.9 years; range, 29–64 years) (P = 0.027). The mean nodule size was 4.6 cm ± 2.7 (standard deviation; range, 0.5–12.7 cm). The mean diameter of the malignant nodules (mean diameter, 5.0 cm ± 3.1; size range, 0.5–12.7 cm) did not significantly differ from that of the benign nodules (mean diameter, 3.9 cm ± 2.3; size range, 0.9–10.1 cm) (P = 0.091). The depth, echogenicity, shape, and margin of the malignant nodules did not significantly differ from those of the benign nodules (Table 1). We found no significant differences in the numbers of nodules or the level of fatty liver (Table 1).

**Figure 4:**
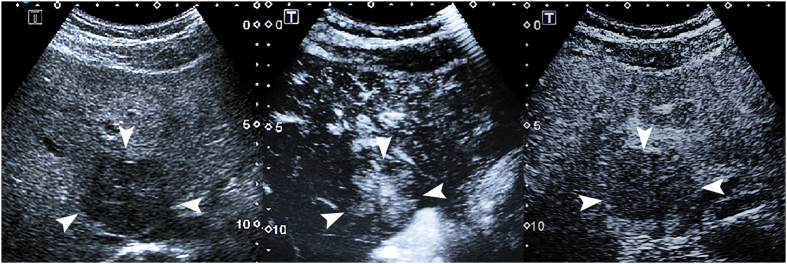
Focal nodular hyperplasia in a 29-year-old man. (**a**) A 5.4-cm lesion (arrows) in the liver shows hypoechogenicity on conventional ultrasound. (**b**) Contrast- enhanced ultrasound shows spoke-wheel arteries (arrow) in the lesion 12 s after contrast agent administration. (**c**) The lesion (arrows) shows hypoenhancement in comparison with adjacent liver tissue 69 s after contrast agent administration.

**Figure 5:**
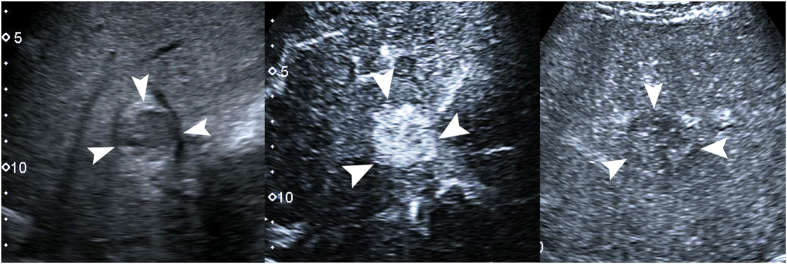
Hepatocellular carcinoma in a 74-year-old man. (**a**) A 2.9-cm lesion (arrows) in the liver shows hypoechogenicity on conventional ultrasound. (**b**) Contrast- enhanced ultrasound shows hyperenhancing (arrow) in the lesion 32 s after contrast agent administration. (**c**) The lesion (arrows) shows hypoenhancement in comparison with adjacent liver tissue 139 s after contrast agent administration.

The emergence phase of the washout time of the malignant nodules did not significantly differ from that of the benign nodules (Table 1). A predominantly different enhancement pattern during the arterial phase was observed in the benign versus the malignant nodules (P < 0.001) (Table 1). Despite the statistical significance, heterogeneous hyperenhancement was found in 18.5% of the benign nodules and in 46.4% of the malignant nodules (P = 0.014). Benign hyperenhancement was found in 44.4% of the benign nodules (Fig. 4) and in none of the malignant nodules (P < 0.001). Homogeneous hyperenhancement and malignant hyperenhancement (Fig. 5) were not found to be significantly different (P = 0.511 and P = 0.132, respectively).

### Quantitative analysis with contrast-enhanced US

Table 2 shows a comparison of the quantitative parameters of the malignant lesions with those of the benign lesions. The PI of the malignant lesions was larger than that of the benign lesions (P = 0.001). The washout time and wash-in continue time of the benign lesions were longer than those of the malignant lesions (P = 0.012 and P = 0.010, respectively). The mean value of the wash-in time interval of the malignant lesions was negative, whereas that of the benign lesions was positive (P = 0.036). There were no significant differences among the wash-in time to the lesion, the wash-in time to the reference and the TTP between the malignant and benign lesions.

### Multiple logistic regression analysis for the quantitative parameters and the contrast-enhanced US features

As in the previous analysis, four quantitative parameters (PI, washout time, wash-in continue time and wash-in time interval) and one contrast-enhanced US feature (enhancement patterns during the arterial phase) were found to be significantly different between the benign and malignant nodules. Table 3 shows the multiple logistic regression analysis for these five variables, the purpose of which was to determine whether the variables were independent predictors of malignancy. The PI, wash-in time interval and enhancement patterns during the arterial phase showed significant associations with the malignant FLLs (P < 0.05). The washout time and wash-in continue time showed no significant associations with the malignant FLLs (P > 0.05).

### Inter-observer variability analysis of quantitative analysis

To test inter-observer variability between the two operators (Q.Y.S. and G.J.L.) for the consequent calculation of quantitative parameters, we compared the results obtained in all of the 83 patients. The ICCs for the seven quantitative parameters ranged from 0.874 to 0.994 (Table 4), indicating excellent agreement between the two observers.

### Diagnostic performance of the contrast-enhanced US and quantitative analysis

No single quantitative parameter suggestive of malignancy had an overall diagnostic accuracy that exceeded 0.700 (Table 5). Criterion set 1 showed a relatively high sensitivity (100%) but a low specificity (44.4%). Criterion set 2 showed a relatively high sensitivity (80.4%) and specificity (88.9%). Criterion set 3 showed a relatively high sensitivity (90.2%) but relatively low specificity (66.7%), with a relatively high AUC (0.845). The AUC of criterion set 2 was significantly higher than that of criterion set 1 (0.904 versus 0.792, P = 0.008) (Table 6).

## Discussion

On contrast-enhanced US, the typical enhancement feature of malignant FLLs appear as hyperenhancing during the arterial phase, followed by washout during the portal venous and late phases^1^,^2^. However, of the 125 benign FLLs with fatty liver background in our study, 33 (26.4%) appeared to have malignant-like enhancements. According to a previous study, the enhanced duration of the liver parenchyma background in fatty liver (300.61 ± 17.42 s) was significantly longer than that in normal liver (213.30 ± 11.84 s, P < 0.05)^7^. That finding implies that a contrast agent persists more markedly and longer in fatty liver tissues compared with in normal liver tissues. We presume that slow contrast agent washout in fatty livers may be one reason for the hypo-enhanced appearance of these benign FLLs during the late phase. However, the signal intensity of fatty liver is significantly higher than in normal liver in conventional US^22^. Thus, the harmonic signals from fatty liver parenchyma may be another possible explanation for the changes in the typical iso-enhancement of benign FLLs.

In addition, FLLs can be characterized based on typical features during the arterial phase, such as chaotic vessels or non-enhancing areas for HCC, rim-enhancement for metastasis, spoke-wheel arteries for FNH, enhanced septa for abscesses, and peripheral nodules for hemangiomas^1^,^20^,^23^,^24^,^25^,^26^,^27^,^28^,^29^,^30^. In the present study, only 9% of the malignant and 44% of the benign FLLs were detected to have specific features. The multiple linear regression analysis showed that the typical features during the arterial phase were significantly associated with the differentiation of FLLs. Although lesions diagnosed according to routine CEUS features were useful in the characterization of FLLs in fatty liver (sensitivity, 100.0%; specificity, 44.4%; and AUC, 0.792), these values are lower than those in normal liver, as has previously been reported^1^,^23^,^24^. Therefore, the characterization of FLLs becomes more difficult in a fatty liver background.

It has been demonstrated that the quantitative parameters of contrast-enhanced US correlate with tumor perfusion and improve the diagnosis of FLLs^8^,^30^,^31^,^32^. With regard to the quantitative parameters, the PI parameter can provide information regarding lesion blood volume and blood flow, and it is related to an abundance of lesion perfusion. In this study, the PI of malignant FLLs was significantly higher than that of benign FLLs, both in the descriptive statistics and in the multiple logistic regression analysis. This result differs from a study reporting FLLs in a normal liver background^31^, which found no significant difference between the PIs of malignant FLLs and benign FLLs. The TTP parameter and the wash-in time interval parameter were associated with the speed of contrast fill-in. There was no significant difference in the TTP between the malignant FLLs and the benign FLLs in the present study. However, the contrast agent wash-in to the malignant FLLs was quicker compared with the reference (i.e., the wash-in time interval was negative). However, for the benign FLLs, the contrast agent wash-in was slower compared with the reference (i.e., the wash-in time interval was positive) (P < 0.05 both in the descriptive statistics and in the multiple logistic regression analysis), suggesting that the contrast agent wash-in time of malignant FLLs is quicker than that of benign FLLs. The washout time parameter and wash-in continue time parameter are related to the speed of the contrast agent washout. In the descriptive statistics, the washout and wash-in continue times for the malignant FLLs were shorter than those for the benign FLLs (P < 0.05), suggesting that the contrast agent washout of malignant FLLs is quicker than that of benign FLLs. However, in the multiple linear regression analysis, there was no significant difference in these two parameters between the malignant FLLs and the benign FLLs, which may suggest that the washout and wash-in continue times are non-independent influencing factors.

In our study, the combination of four significant quantitative parameters (PI, wash-in time interval, washout time and wash-in continue time) with routine contrast-enhanced US features to predict malignant FLLs versus benign FLLs performed superior to the use of routine contrast-enhanced US features, as did the diagnostic performance of criterion set 2 (sensitivity, 80.4%; specificity, 88.9%) compared with criterion set 1 (sensitivity, 100.0%; specificity, 44.4%). Quantitative evaluation of contrast-enhanced US substantially improves the differential diagnosis of atypical benign FLLs from malignant lesions in fatty liver, with an AUC improved from 0.792 to 0.904 (P = 0.008). Furthermore, for FLLs that were impossible to characterize using any specific contrast-enhanced US features (66/83) (Fig. 3), we also achieved an adequate diagnostic accuracy (up to 0.845) (Table 6) using parametric features for these FLLs (criterion set 3). In addition, using parametric features in these 66 atypical lesions improved the diagnosis by 84.8% (56/66) (Fig. 1).

The present study had some limitations. First, the final diagnoses of fatty liver and FLLs were not totally confirmed based on pathology due to ethical concerns. However, we believe that based on patient history, laboratory data, multiple imaging findings, and follow-ups, the final diagnoses of fatty liver and FLLs should be sufficiently appropriate according to well-established clinical diagnostic criteria. Second, the case number was relatively small, and this is a retrospective review, which is fraught with some bias. Further prospective studies with larger numbers of cases are necessary. Third, several factors, such as cardiac output, contrast injection rate and volume, local resistance to flow, the type of lesion may affect the value of US parameters, and a well-designed prospective study considering all the possible influential factors to US parameters is necessary. Finally, how the liver background affects the diagnostic performance of FLLs was only discussed by comparing our results with published studies. Further studies focused on a quantitative comparison of the FLLs between cirrhosis and non-cirrhotic livers, and among different fatty liver levels are needed. In addition, a control group of patients with liver focal lesions without excessive fatty infiltration should be included.

In conclusion, the quantitative analysis of contrast-enhanced US images improves the diagnostic performance of the differentiation of atypical benign FLLs from malignancies in a fatty liver background.

## Additional Information

**How to cite this article**: Shan, Q.-Y. *et al.* Focal Lesions in Fatty Liver: If Quantitative Analysis Facilitates the Differentiation of Atypical Benign from Malignant Lesions. *Sci. Rep.* **6**, 18640; doi: 10.1038/srep18640 (2016).

## Figures and Tables

**Table 1:** Clinical Characteristics, Conventional US and contrast-enhanced US Characteristics of the Benign and Malignant FLLs.

Characteristics	Malignant	Benigh FLLs(n = 27)	*P* value
Age (Mean ± SD year)	52 ± 13	46 ± 10	0.027
Male/female (n)	44/12	18/9	0.242
Weight (kg)	68 ± 6	70 ± 8	0.160
Viral hepatitis (with/without) (n)	22/34	2/25	0.003
Size (Mean ± SD mm)	50 ± 31	39 ± 23	0.091
Depth (Mean ± SD mm)	82 ± 27	66 ± 21	0.333
Number of nodules, n (%)			0.315
Single	38(68)	22(81)	
Multiple	18(32)	5(19)	
Echogenicit, n (%)			0.514
Hypoechogenicity	32(57)	19(70)	0.246
Isoechogenicity	2(4)	0(0)	0.320
Hyperechogenicity	5(9)	1(4)	0.389
Mixed echoic	17(30)	7(26)	0.677
Shape, n (%)			0.616
Round	30(54)	13(48)	0.643
Ovoid	6(11)	5(19)	0.326
Irregular	20(35)	9(33)	0.831
Margin, n (%)			0.199
Well-defined	41(73)	16(59)	
Ill-defined	15(27)	11(41)	
Level of fatty liver, n (%)			0.077
I	38(68)	13(48)	0.069
II	14(25)	10(37)	0.190
III	4(7)	4(15)	0.233
Emergence phase of washout time, n (%)			0.752
portal phase (≤120 s)	51(91)	24(89)	
late phase (>120 s)	5(9)	3(11)	
Enhancement patterns during arterial phase, n (%)			<0.001
Homogeneous hyperenhancement	25(45)	10(37)	0.511
Heterogeneous hyperenhancement	26(46)	5(19)	0.014
Malignant contrast-enhanced US features	5(9)	0(0)	0.132
Benign contrast-enhanced US features	0(0)	12(44)	<0.001

Note.—Unless otherwise indicated, the data are the number of nodules, with percentages in parentheses.

The diagnostic features for malignant lesions included the following: rim-enhancement, and/or with complete enhancement, hyperenhancement, or nonenhancing regions; hyperenhancing, and/or with nonenhancing regions; rim-like hyperenhancement, central hypoenhancement, and/or with nonenhancing regions, inhomogeneous, or hyperenhancement; hyperenhancing, complete nonenhancing areas (if large), and/or with enhancing tumor thrombus or hypo/nonenhancing during the arterial phase. The diagnostic features for benign lesions included the following: peripheral nodular enhancement, or small lesion with complete, rapid centripetal enhancement; hyperenhancing from the center, complete, early, and/or with spoke-wheel arteries or feeding artery; hyperenhancing, complete, and/or with nonenhancing regions; isoenhancing; peripheral enhancement, no central enhancement, and/or with enhanced septa or hyperenhanced liver segment during the arterial phase.

**Table 2:** Quantitative parameters of the contrast-enhanced US.

Quantitative Parameter *	Malignant FLLs			Benign FLLs			*P*value
	Range	Mean	Standard Deviation	Range	Mean	Standard Deviation
wash-in time in the lesion	7–19	13.77	2.69	8–19	13.37	2.96	0.543
wash-in time in reference	8–19	14.50	2.96	5–19	13.00	3.35	0.053
wash-in time interval	−4–6	−0.68	1.51	−2–6	0.37	1.71	0.036
PI	1.03–4.35	1.75	0.61	1.02–2.29	1.40	0.36	0.001
TTP	15–45	26.41	6.27	17–47	25.56	7.27	0.603
wash-out time	21–193	65.11	36.70	25–174	87.04	36.08	0.012
wash-in continue time	8–176	51.34	36.03	12–158	73.7	36.34	0.010

*PI = peak intensity, TTP = time to peak.

**Table 3:** Results of the Multiple Logistic Regression Analysis for the quantitative parameters and the contrast-enhanced US features.

Quantitative Parameters andCEUS features*	β Coefficient	Standard Errors	*P* Value
PI	2.02	0.86	0.018
wash-out time	0.05	0.11	0.676
wash-in continue time	−0.07	0.11	0.548
wash-in time interval	−0.35	0.17	0.036
Enhancement patterns during arterial phase	−0.85	0.32	0.007

*PI = peak intensity.

**Table 4:** Inter-observer variability of quantitative analysis.

Observers	ICC (95% CI)						
	wash-in time in thelesion	wash-in time inreference	wash-in timeinterval	PI	TTP	wash-out time	wash-in continuetime
Radiologist (G.J.L.) vs. Radiologist (Q.Y.S.)	0.951 (0.926–0.968)	0.970 (0.954–0.980)	0.876 (0.815–0.918)	0.874 (0.805–0.918)	0.959 (0.937–0.973)	0.994 (0.990–0.996)	0.993 (0.990–0.996)

*ICC = intraclass correlation coefficient, PI = peak intensity, TTP = time to peak.

Note.—Numbers in parentheses are 95% confidence intervals.

**Table 5:** Diagnostic Accuracy of the Quantitative Parameters of contrast-enhanced US.

Quantitative Parameters*	Sensitivity(%)	Specificity(%)	PPV(%)	NPV(%)	AUC	Cut-offValue
wash-in time in the lesion	60.71	59.26	75.6	42.1	0.560	>13
wash-in time in reference	64.29	62.96	78.3	45.9	0.634	>13
wash-in time interval	89.29	48.15	78.1	68.4	0.688	< = 0
PI	62.5	77.78	85.4	50	0.690	>1.4677
TTP	80.36	40.74	73.8	50	0.561	>21
wash-out time	66.07	74.07	84.1	51.3	0.690	< = 69
wash-in continue time	69.64	74.07	84.8	54.1	0.691	< = 58

*PI = peak intensity, TTP = time to peak, PPV = positive predictive value, NPV = negative predictive value, AUC = area under the receiver operating characteristic curve.

**Table 6:** Diagnostic Performances of the Three Criterion Sets for FLLs.

Criteria*	Sensitivity (%)	Specificity (%)	PPV (%)	NPV (%)	AUC
Criterion 1	100	44.44	78.9	100	0.792
Criterion 2	80.36	88.89	93.7	68.6	0.904 (0.0078)
Criterion 3	90.2	66.67	90.2	66.7	0.845

Note.—The data in parentheses are the *P* values in comparison with **c**riterion set 1.

*Criterion 1 = lesions diagnosed according to routine contrast-enhanced US features; **c**riterion 2 = parametric features added to **c**riterion set 1; **c**riterion 3 = liver lesions that could not be diagnosed using contrast-enhanced US but instead were diagnosed using parametric features.
